# Associations of obesity-related indices with prediabetes regression to normoglycemia among Chinese middle-aged and older adults: a prospective study

**DOI:** 10.3389/fnut.2023.1075225

**Published:** 2023-05-19

**Authors:** Hongguang Yang, Minjie Zhang, Jiaqi Nie, Minzhe Zhang, Gaolei Lu, Rui Chen, Qiqiang He

**Affiliations:** ^1^School of Public Health, Wuhan University, Wuhan, China; ^2^Hubei Biomass-Resource Chemistry and Environmental Biotechnology Key Laboratory, Wuhan University, Wuhan, China

**Keywords:** obesity, obesity-related indices, prediabetes regression, abdominal fat, insulin resistance

## Abstract

**Background:**

Prediabetes is associated with increased cardiovascular risk and all-cause mortality, while its regression will decrease the risks. This study investigated the associations of six obesity-related indices (waist-to-height ratio (WHtR), body roundness index (BRI), conicity index (CI), body shape index (ABSI), Chinese visceral adiposity index (CVAI), and triglyceride glucose (TyG) index) with prediabetes regression based on the China Health and Retirement Longitudinal Study (CHARLS), enrolling middle-aged and older adults.

**Methods:**

We included 2,601 participants with prediabetes from CHARLS, who were followed up from 2011–2012 to 2015–2016, with blood samples collected for measuring fasting plasma glucose and hemoglobin A1c. All the obesity-related indices at baseline and their dynamic changes were calculated and categorized into tertiles. Logistic regression analysis was applied to obtain the odds ratio (OR) and 95% confidence intervals (CIs). Attributable fractions (AFs) and 95% CIs of these indices and the dynamic changes were calculated with the AF package in R software, and the cutoff values of initial obesity-related indices were obtained by the receiver operating characteristic (ROC) analysis.

**Results:**

During the 4-year follow-up period, 562 (21.61%) participants regressed from prediabetes to normoglycemia. They had lower initial BRI, WHtR, CI, ABSI, CVAI, and TyG than those who did not (*P* < 0.05). After multivariable adjustment, participants in the first tertile of initial BRI (OR, 1.45, 95%CIs, 1.09–1.93), WHtR (OR, 1.46, 95%CIs, 1.10–1.95), and CVAI (OR, 1.47, 95%CIs, 1.11–1.93) had increased odds of prediabetes regression compared with those in the highest tertile. Participants with decreased TyG (OR, 2.08; 95%CIs, 1.61–2.70) also had increased odds of prediabetes regression compared with those with increased TyG. The cutoff values of initial obesity-related indices were 4.374 for BRI, 0.568 for WHtR, 8.621 for TyG, 1.320 for CI, 0.083 for ABSI, and 106.152 for CVAI, respectively. The AFs were 21.10% for BRI < 4.374, 20.85% for WHtR < 0.568, 17.48% for CVAI < 107.794, and 17.55% for ΔTyG < 0, respectively.

**Conclusion:**

Low initial BRI, WHtR, and CVAI, as well as TyG reduction, were significantly related to prediabetes regression to normoglycemia, and the AFs were around 20%. Less abdominal fat and insulin resistance reduction would benefit future health outcomes among people with prediabetes.

## Introduction

Prediabetes, including impaired fasting glucose (IFG) and impaired glucose tolerance (IGT), is defined as a blood glucose level higher than normal but lower than the thresholds of diabetes (1–3). Globally, there are around 374 million people living with IGT, and there will be more than 470 million people with IGT by 2030 (4, 5). In China, the estimated prevalence of prediabetes was 35.2% among Chinese adults between 2015 and 2017 (6). Prediabetes has been associated with a significantly increased risk of cardiovascular diseases, chronic kidney disease, cancer, and all-cause mortality (7, 8), while the regression to normoglycemia could reduce the risks (9). Therefore, it is of crucial importance to identify modifiable factors to aid in regressing from prediabetes to normoglycemia.

Several factors such as body weight loss, structured physical activity, and metformin use have been associated with prediabetes regression, while obesity may be detrimental to this process (7, 10–14). Obesity is considered a major risk factor for prediabetes and diabetes, while its role in prediabetes regression to normoglycemia is still mixed. The KORA F4 cohort study found the reduction of body mass index (BMI) and waist circumference (WC), instead of initial BMI and WC, contributed to the reversing from prediabetes, defined by hemoglobin A1c (HbA1c), to normal glucose tolerance (NGT) in older Germany adults (14, 15). Nevertheless, Kowall et al. reported that the reduction of BMI and WC had no effect on glucose-defined prediabetes regression (15). Evidence suggested that BMI and WC have some drawbacks to evaluate obesity and metabolism abnormality (16, 17). BMI can only identify general obesity (17), while WC could identify abdominal obesity, but it does not account for differences in body height (16, 17).

Waist-to-height ratio (WHtR), body roundness index (BRI), conicity index (CI), body shape index (ABSI), and Chinese visceral adiposity index (CVAI) are surrogate markers for abdominal obesity and have been shown to be more correlated with metabolic abnormality than BMI and WC (17–19). A Chinese cohort study suggested BRI and WHtR as the best anthropometric indices for predicting diabetes risk (20). However, no prior study has explored the relationship between these indices and prediabetes regression. Meanwhile, the triglyceride glucose (TyG) index is also a marker of obesity and is highly associated with insulin resistance (IR) (21). Previous studies indicated that TyG was more suitable for the determination of IR than HOMI-IR and had a great ability to identify prediabetes and diabetes (22, 23). However, its association with prediabetes regression to normoglycemia is still unclear.

Although several obesity-related indices have been associated with prediabetes and diabetes, studies investigating these indices in relation to prediabetes regression are lacking. Therefore, this study aimed to investigate the associations of obesity-related indices and their dynamic changes with prediabetes regression to normoglycemia in middle-aged and older adults from the China Health and Retirement Longitudinal Study (CHARLS).

## Methods

### Study population

The present study used data from the CHARLS, which enrolled a nationally representative sample of community dwellers aged ≥45 years in China. The design of CHARLS has been described in detail elsewhere (24). A total of 17,708 participants from 450 urban communities and rural areas in 28 provinces of China were recruited from 2011 to 2012 (Wave 1). They were followed up every 2 years, and there were three subsequent follow-ups. In this study, data from Wave 1 and Wave 3 (2015–2016) were used because blood samples were only collected in the two waves.

**Figure 1:**
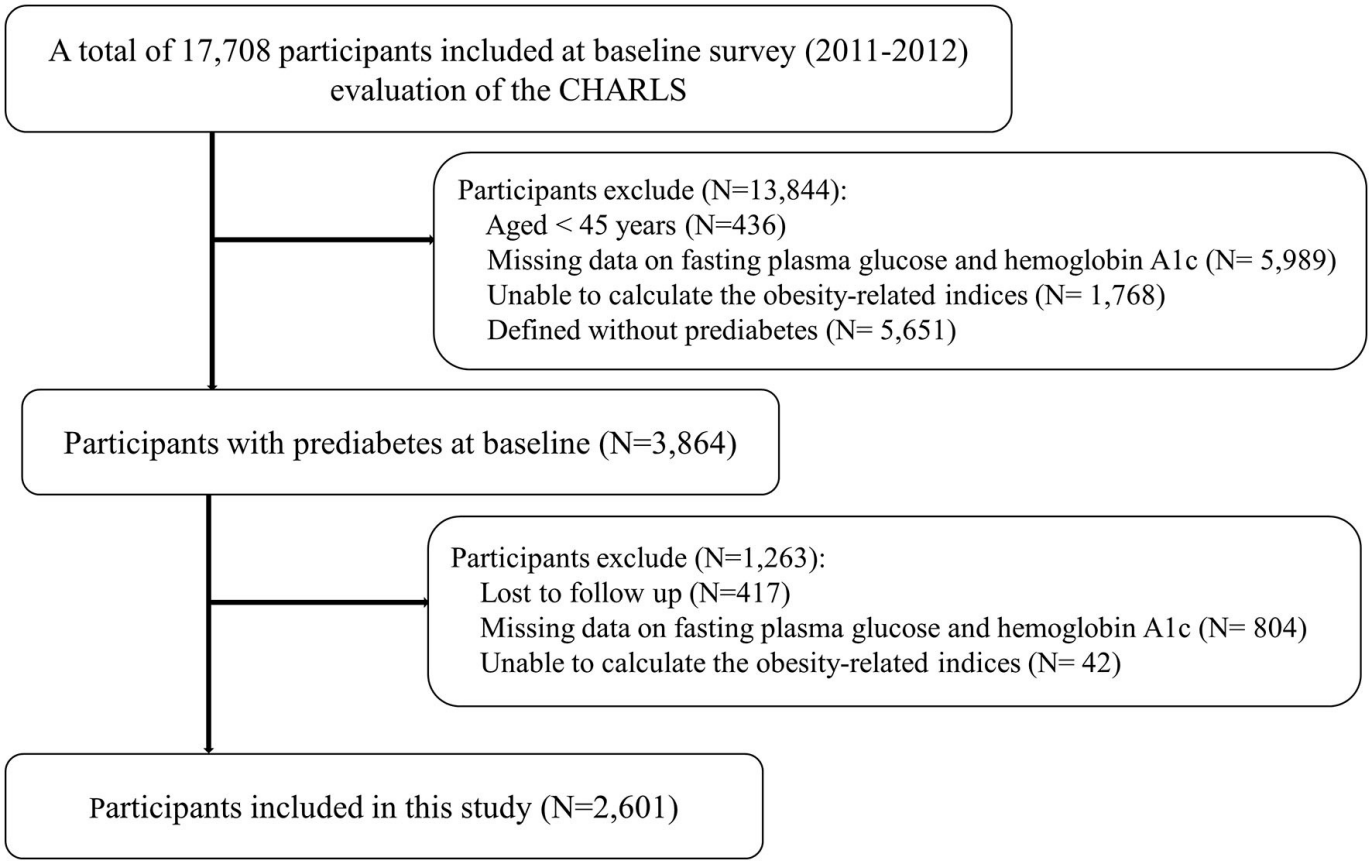
Study flow chart.

Of 17,708 participants, we excluded 436 participants aged <45 years, 5,989 participants with missing data on fasting plasma glucose (FPG) and HbA1c, 1,768 participants unable to calculate obesity-related indices, and 5,651 participants without prediabetes. At the follow-up, we further excluded 417 participants who lost to follow-up, 804 participants with missing data on FPG and HbA1c, and 42 participants unable to calculate these indices. Finally, a total of 2,601 participants were included in the present study. Details regarding the study population selection are presented in Figure 1.

### Data collection

Fasting blood samples were collected for measurements of FPG, HbA1c, total cholesterol (TC), triglycerides (TG), high-density lipoprotein-cholesterol (HDL-c), and low-density lipoprotein-cholesterol (LDL-c). FPG, TG, TC, HDL-c, and LDL-c were measured by an enzymatic colorimetric test method, whereas the HbA1c assay was performed by the boronate affinity high-performance liquid chromatography (HPLC) method. Height and weight were measured via standard methods, with participants wearing light clothes without shoes. WC was measured to the nearest ± 0.5 cm at the minimum circumference between the lowest ribs. BMI was calculated by dividing weight (kg) by height squared (m^2^). Obesity-related indices were calculated by following formulas (19, 25) and were categorized into tertiles.


$$\begin{array}{l} \text{BRI}=364.2-365.5\times {\left[1-\pi^{-2}{\text{WC}}_{\left(\text{m}\right)}^{2}{\text{height}}^{-2}{\;}_{\left(\text{m}\right)}\right]}^{1/2} \end{array}$$

$$\begin{array}{l} \text{WHtR}=\;{\text{WC}}_{\left(\text{cm}\right)}/{\text{height}}_{\left(\text{cm}\right)} \end{array}$$

$$\begin{array}{l} \text{TyG}=\text{ln}\left({\text{TG}}_{\left(\text{mg}/\text{dL}\right)}\times \;{\text{FPG}}_{\left(\text{mg}/\text{dL}\right)}/2\right) \end{array}$$

$$\begin{array}{l} \text{CI}=\left[{\text{WC}}_{\left(\text{m}\right)}\right]\times 0.109^{-1}\times {\left[{\text{weight}}_{\left(\text{kg}\right)}/{\text{height}}_{\left(\text{m}\right)}\right]}^{-1/2} \end{array}$$

$$\begin{array}{l} \text{ABSI}=\left[{\text{WC}}_{\left(\text{m}\right)}\right]{\text{BMI}}^{-2/3}{\text{height}}_{\left(\text{m}\right)}^{-1/2} \end{array}$$

$$\begin{array}{l} \text{CVAI: CVAI}=-267.93+0.68\times \text{age}+0.03\times \text{BMI}+4\times \text{WC}\\ +22\times \text{log }{\text{TG}}_{\left(\text{mmol}/\text{l}\right)}-16.32\times {\text{HDL}-\text{c}}_{\left(\text{mmol}/\text{l}\right)}\text{ in males} \end{array}$$

$$\begin{array}{l} \text{CVAI}=-187.32+1.71\times \text{age}+4.23\times \text{BMI}+1.12\times \text{WC}\\ +39.76\times {\text{logTG}}_{\left(\text{mmol}/\text{l}\right)}-11.66\times {\text{HDL}-\text{c}}_{\left(\text{mmol}/\text{l}\right)}\text{ in females}. \end{array}$$

Dynamic changes in the indices were calculated by subtracting the baseline values from those of the follow-up and were categorized into tertiles (decreased, stable, and increased).

### Definition of prediabetes, diabetes, and normoglycemia

According to the criteria of the American Diabetes Association (7), prediabetes was defined as FPG in the range of 5.6–6.9 mmol/L or HbA1c in the range of 5.7–6.4%; normoglycemia was as FPG < 5.6 mmol/L and HbA1c < 5.7%; and diabetes was as FPG ≥ 7.0 mmol/L, HbA1c ≥ 6.5%, self-reported history, and/or the use of anti-diabetic medications.

### Covariates

Participants reported their sociodemographic characteristics, including age, gender, education level (primary school or below, middle school, and high school or above), place of residence, marital status, and smoking and drinking history. Hypertension was determined by either clinical diagnosis or self-reported hypotensive treatment or SBP ≥ 140 mmHg or DBP ≥ 90 mmHg. Dyslipidemia was defined as TC ≥ 6.22 mmol/L, TG ≥ 2.26 mmol/L, HDL-c < 1.04 mmol/L, LDL-c ≥ 4.14 mmol/L, or self-reported dyslipidemia (26, 27). Additionally, TG, TC, LDL-c, HDL-c, FPG, and HBA1c at the baseline were included in confounders.

### Statistical analysis

The normality of continuous variables was inspected visually by QQ-plot, which suggested no serious violations of normality assumptions. Continuous variables in our study are presented as means ± standard deviations, and the two groups were compared using the *t*-test. Categorical variables are presented as frequency (*n*, %), and the two groups were compared using the chi-square test. Multiple logistic regression analysis was conducted to assess the association of obesity-related indices and their dynamic changes with regression to normoglycemia. According to previous research (7), three different models were introduced as follows: Model 1, without adjustment; Model 2, adjusted for age and gender; and Model 3, further adjusted for the covariates including place of residence, marital status, educational level, history of smoking and drinking, presence of hypertension and dyslipidemia, SBP, DBP, TG, TC, LDL-c, HDL-c, FPG, and HbA1c at the baseline.

Subgroup analyses were performed to evaluate the association between obesity-related indices and prediabetes regression stratified by age. In the sensitivity analysis, we used the 1999 World Health Organization (WHO) criteria for diabetic status to assess the association of obesity-related indices with the regression of prediabetes (28). Prediabetes was defined as FPG 6.1–6.9 mmol/L; normoglycemia was defined as FPG < 6.1 mmol/L, and diabetes was defined as FPG ≥ 7.0 mmol/L, HbA1c ≥ 6.5%, self-reported history, and/or the use of anti-diabetic medications. In addition, we further performed sensitivity analysis based on multinomial logistic regression in which three outcome groups were taken into consideration over follow-up: regression to normoglycemia, progression to diabetes, and remained as prediabetes and progression to diabetes group was used as the reference.

Population attributable fraction (PAF) and attributable fraction (AF) are the embodiments of the percentage reduction of a given outcome that is expected if there is no exposure (29). The details of AF and the formula were described elsewhere (30). AFs and 95%CI were calculated with the AF package in R software, which allows for confounder-adjusted estimation of AFs for cohort studies (30). Here, we defined the exposure as low levels of initial obesity-related indices and their reduction to obtain the AFs. To calculate AFs, we obtained the cutoff values of initial obesity-related indices by the receiver operating characteristic (ROC) analysis. Then, we defined the initial values lower than the corresponding cutoff values as the exposure and the dynamic change lower than 0 as the exposure in AF analysis.

Pearson's correlation analysis was performed to assess the correlations between obesity-related indices, fasting glucose, and HbA1c.

All statistical analyses were performed using SPSS version 24.0 (IBM Corp., Armonk, NY) and R language environment (version 3.3.1, http://www.r-project.org). A two-sided *p*-value of < 0.05 was considered statistically significant.

## Results

### Baseline characteristics

A total of 2,601 participants with prediabetes at the baseline were included in this study. The basic characteristics are shown in Table 1. The mean age (SD) of participants was 59.34 (8.67) years old, and 1,194 (45.9%) participants were men. During the 4-year follow-up period, 562 participants regressed to normoglycemia. They showed a lower prevalence of dyslipidemia and lower initial SBP, WC, BMI, TG, TC, LDL, FPG, and HbA1c (*P* < 0.05) than those who did not regress to normoglycemia. Participants who regressed to normoglycemia also had lower BRI, WHtR, TyG, CI, ABSI, and CVAI (*P* < 0.05) than participants who did not regress to normoglycemia.

**Table 1:** Baseline characteristics of participants stratified by glycemic condition.

**Characteristic**	**Total**	**Regression to normoglycemia**		***P***
		**No**	**Yes**	
No. of subjects	2,601	2,039	562	
Age, years	59.34 ± 8.67	59.69 ± 8.62	58.21 ± 8.76	< 0.001
Male, *n* (%)	1,195 (45.9)	904 (44.3)	291 (51.8)	0.002
Place of residence, *n* (%)				0.743
Rural	2,175 (83.6)	1,702 (83.5)	473 (84.2)	
Urban	426 (16.4)	337 (16.5)	89 (15.8)	
Marital status, *n* (%)				0.982
Married	2,274 (87.4)	1,728 (84.7)	492 (87.5)	
Not married	2,274 (12.6)	311 (15.3)	70 (12.5)	
Education level				0.035
Primary school or below	1,858 (71.4)	1,479 (72.5)	379 (67.4)	
Middle school	537 (20.6)	410 (20.1)	127 (22.6)	
High school or above	206 (7.9)	150 (7.4)	56 (10.0)	
Smoking, *n* (%)	1,615 (62.1)	1,277 (62.6)	338 (60.1)	0.344
Drinking, *n* (%)	845 (32.5)	647 (31.7)	198 (35.2)	0.118
Chronic diseases history				
Hypertension, *n* (%)	1,097 (42.2)	878 (43.1)	219 (39.0)	0.090
Dyslipidemia, *n* (%)	941 (36.2)	758 (37.2)	183 (32.6)	0.049
WC (cm)	85.07 ± 12.42	85.63 ± 12.42	83.03 ± 12.25	< 0.001
SBP (mmHg)	131.46 ± 24.52	131.83 ± 23.35	130.12 ± 28.31	0.004
DBP (mmHg)	76.17 ± 11.96	76.34 ± 11.88	75.58 ± 12.23	0.138
TC (mmol/L)	5.10 ± 0.99	5.15 ± 0.99	4.91 ± 0.99	< 0.001
TG (mmol/L)	1.52 ± 0.97	1.52 ± 0.95	1.49 ± 1.05	0.045
LDL-c (mmol/L)	3.09 ± 0.93	3.14 ± 0.93	2.91 ± 0.91	< 0.001
HDL-c (mmol/L)	1.33 ± 0.40	1.33 ± 0.40	1.33 ± 0.40	0.819
FPG (mmol/L)	6.02 ± 0.37	6.03 ± 0.38	5.99 ± 0.34	0.002
HbAlc (%)	5.20 ± 0.42	5.25 ± 0.42	5.01 ± 0.39	< 0.001
BMI	23.80 ± 3.84	23.93 ± 3.85	23.36 ± 3.80	< 0.001
BRI	4.26 ± 1,52	4.35 ± 1.54	3.94 ± 1.38	< 0.001
WHtR	0.54 ± 1.31	0.54 ± 0.08	0.52 ± 0.08	< 0.001
TyG	8.74 ± 0.55	8.75 ± 0.54	8.70 ± 0.57	0.023
CI	1.27 ± 0.14	1.28 ± 0.14	1.26 ± 0.15	< 0.001
ABSI	0.08 ± 0.01	0.08 ± 0.01	0.08 ± 0.01	0.001
CVAI	97.54 ± 43.54	99.59 ± 43.65	90.11 ± 42.34	< 0.001

WC, waist circumference; SBP, systolic blood pressure; DBP, diastolic blood pressure; FPG, fasting plasma glucose; HbA1c, hemoglobin A1c; TG, triglycerides; TC, total cholesterol; LDL-c, low-density lipoprotein-cholesterol; HDL-c, high-density lipoprotein-cholesterol; BMI, body mass index; BRI, body roundness index; WHtR, waist-to-height ratio; TyG, triglyceride glucose index; CI, conicity index; ABSI, a body shape index; CVAI, Chinese visceral adiposity index.

### Initial obesity-related indices at baseline and prediabetes regression

The association of obesity-related indices at baseline with prediabetes regression is presented in Table 2. Initial BRI, WHtR, and CVAI showed a significant association with prediabetes regression. In the unadjusted model (Model 1), compared with the highest tertile, the ORs of the first tertile were 1.99 (95%CIs, 1.57–2.53) for BRI, 2.00 (95%CIs, 1.58–2.55) for WHtR, and 1.79 (95%CIs, 1.42–2.29) for CVAI, respectively. After adjusting for potential confounders (Model 3), the ORs were 1.45 (95%CIs, 1.09–1.93) for BRI, 1.46 (95%CIs, 1.10–1.95) for WHtR, and 1.47 (95%CIs, 1.11–1.93) for CVAI, respectively. However, there was no significant association between initial CI, ABSI, TyG, and prediabetes regression. In addition, elevated WC showed decreased odds of regression from prediabetes to normoglycemia (OR, 0.989; 95%CIs, 0.981–0.998), while there was no significant association between BMI and prediabetes regression to normoglycemia (Supplementary Table S1).

**Table 2:** Initial obesity-related indices and prediabetes regression.

		**Model 1^a^**	**Model 2^b^**	**Model 3^c^**
**Variables**	**No. of cases/total**	**OR (95% CIs)**	**OR (95% CIs)**	**OR (95% CIs)**
**BRI**				
Tertile 1 (< 3.582)	226/866	1.99 (1.57, 2.53)	1.84 (1.43, 2.37)	1.45 (1.09, 1.93)
Tertile 2 (3.582–4.818)	205/867	1.74 (1.37, 2.23)	1.63 (1.27, 2.09)	1.38 (1.06, 1.81)
Tertile 3 (>4.818)	131/868	1 (Ref.)	1 (Ref.)	1 (Ref.)
*P* for trend		< 0.001	< 0.001	0.015
**WHtR**				
Tertile 1 (< 0.512)	226/866	2.00 (1.58, 2.55)	1.86 (1.44, 2.39)	1.46 (1.10, 1.95)
Tertile 2 (0.512–0.572)	206/868	1.76 (1.38, 2.25)	1.65 (1.29, 2.12)	1.40 (1.07, 1.83)
Tertile 3 (>0.572)	130/867	1 (Ref.)	1 (Ref.)	1 (Ref.)
*P* for trend		< 0.001	< 0.001	0.013
**TyG**				
Tertile 1 (< 8.463)	206/866	1.26 (1.00, 1.59)	1.27 (1.00, 1.60)	1.20 (0.84, 1.70)
Tertile 2 (8.463–8.934)	184/868	1.09 (0.86, 1.37)	1.11 (0.88, 1.41)	1.14 (0.84, 1.55)
Tertile 3 (>8.934)	172/867	1 (Ref.)	1 (Ref.)	1 (Ref.)
*P* for trend		0.046	0.046	0.543
**CI**				
Tertile 1 (< 1.254)	216/867	1.67 (1.32, 2.11)	1.46 (1.14, 1.87)	1.19 (0.92, 1.57)
Tertile 2 (1.254–1.324)	202/866	1.53 (1.21, 1.94)	1.37 (1.07, 1.75)	1.20 (0.93, 1.56)
Tertile 3 (>1.324)	144/868	1 (Ref.)	1 (Ref.)	1 (Ref.)
*P* for trend		< 0.001	0.003	0.211
**ABSI**				
Tertile 1 (< 0.081)	203/866	1.30 (1.04, 1.65)	1.11 (0.86, 1.41)	0.96 (0.74, 1.25)
Tertile 2 (0.081–0.085)	194/867	1.23 (0.97, 1.55)	1.09 (0.85, 1.38)	0.79 (0.79, 1.31)
Tertile 3 (>0.085)	165/868	1 (Ref.)	1 (Ref.)	1 (Ref.)
P for trend		0.025	0.435	0.747
**CVAI**				
Tertile 1 (< 81.131)	216/867	1.76 (1.38, 2.23)	1.61 (1.26, 2.05)	1.33 (1.01, 1.77)
Tertile 2 (81.131–114.538)	211/867	1.59 (1.25, 2.01)	1.55 (1.22, 1.97)	1.26 (0.97, 1.64)
Tertile 3 (>114.538)	135/867	1 (Ref.)	1 (Ref.)	1 (Ref.)
*P* for trend		< 0.001	< 0.001	< 0.001

Abbreviations are the same as presented in Table 1; OR, odds ratio; CIs, confidence intervals.

a Unadjusted.

b Adjusted for age and gender.

c Adjusted for age, gender, place of residence, marital status, educational level, history of smoking and drinking, presence of hypertension, dyslipidemia, systolic blood pressure, diastolic blood pressure, triglycerides, total cholesterol, low-density lipoprotein-cholesterol, high-density lipoprotein-cholesterol, fasting plasma glucose, and hemoglobin A1c at baseline (for TyG, except triglycerides and fasting plasma glucose; for CVAI, except triglycerides and high-density lipoprotein-cholesterol).

The sensitivity analyses suggested BRI, WHtR, CI, and CVAI were significantly associated with prediabetes regression (Supplementary Tables S2, S4). In the subgroup analyses, the associations of BRI, WHtR, CI, and CVAI with prediabetes regression to normoglycemia were significant in the subgroups of participants aged ≥60 years, not drinking group, and participants with high HbA1c level. Initial BRI and WHtR in the group aged <60 years and female group, WHtR in the low HbA1c level group, and CVAI in the male group were also significantly associated with prediabetes regression (Supplementary Figures S1, S3, S5, S7).

### Dynamic changes of obesity-related indices with prediabetes regression

The association of dynamic changes of obesity-related indices with prediabetes regression is presented in Table 3. ΔTyG showed a significant association with prediabetes regression to normoglycemia. In the unadjusted model (Model 1), compared with the highest tertile, the OR of the first tertile of ΔTyG was 1.90 (95%CIs: 1.51–2.40). After adjusting for potential confounders (Model 3), the OR was 2.19 (95%CIs: 1.68–2.85). Nevertheless, ΔBRI, ΔWHtR, ΔCI, ΔABSI, and ΔCVAI showed no significant association with prediabetes regression to normoglycemia. In addition, ΔBMI and ΔWC showed no significant association with prediabetes regression to normoglycemia (Supplementary Table S1).

**Table 3:** Dynamic changes in obesity-related indices and prediabetes regression.

		**Model 1^a^**	**Model 2^b^**	**Model 3^c^**
**Variables**	**No. of cases/total**	**OR (95% CIs)**	**OR (95% CIs)**	**OR (95% CIs)**
Δ**BRI**				
Decreased (< -0.188)	185/866	1.00 (0.80, 1.26)	1.04 (0.82, 1.31)	1.14 (0.90, 1.46)
Stable (−0.188 to 0.461)	192/867	1.05 (0.84, 1.32)	1.04 (0.82, 1.30)	1.05 (0.82, 1.34)
Increased (>0.461)	185/868	1 (Ref.)	1 (Ref.)	1 (Ref.)
*P* for trend		0.98	0.766	0.284
Δ**WHtR**				
Decreased (< -0.009)	187/867	1.00 (0.80, 1.26)	1.03 (0.82, 1.30)	1.14 (0.89, 1.45)
Stable (−0.009 to 0.023)	188/867	1.01 (0.80, 1.27)	1.00 (0.79, 1.26)	1.02 (0.80, 1.29)
Increased (>0.023)	187/867	1 (Ref.)	1 (Ref.)	1 (Ref.)
P for trend		0.998	0.776	0.307
Δ**TyG**				
Decrease (< -0.214)	238/867	1.90 (1.51, 2.40)	1.90 (1.51, 2.41)	2.08 (1.61, 2.70)
Stable (−0.214 to 0.224)	180/867	1.32 (1.03, 1.68)	1.35 (1.06, 1.73)	1.37 (1.06, 1.77)
Increased (>0.224)	144/867	1 (Ref.)	1 (Ref.)	1 (Ref.)
*P* for trend		< 0.001	< 0.001	< 0.001
Δ**CI**<**0**				
Decreased (< -0.025)	174/867	0.95 (0.75, 1.2)	0.98 (0.77, 1.24)	1.03 (0.81, 1.32)
Stable (−0.025 to 0.038)	207/867	1.19 (0.95, 1.49)	1.15 (0.92, 1.45)	1.15 (0.91, 1.46)
Increased (>0.038)	181/867	1 (Ref.)	1 (Ref.)	1 (Ref.)
*P* for trend		0.683	0.876	0.783
Δ**ABSI**<**0**				
Decreased (< -0.002)	171/866	0.89 (0.7, 1.12)	0.91 (0.72, 1.15)	0.74 (0.74, 1.21)
Stable (−0.002 to 0.002)	203/868	1.1 (0.88, 1.38)	1.06 (0.84, 1.33)	1.02 (0.80, 1.30)
Increased (>0.002)	188/867	1 (Ref.)	1 (Ref.)	1 (Ref.)
*P* for trend		0.327	0.437	0.664
Δ**CVAI**<**0**				
Decreased (< -1.367)	191/867	1.06 (0.85, 1.34)	1.10 (0.87, 1.38)	1.18 (0.93, 1.51)
Stable (−1.367 to 15.666)	184/867	0.97 (0.77, 1.22)	1.02 (0.81, 1.29)	1.02 (0.80, 1.31)
Increased (>15.666)	187/867	1 (Ref.)	1 (Ref.)	1 (Ref.)
*P* for trend		0.861	0.597	0.275

OR, odds ratio; CIs, confidence intervals; ΔBRI, dynamic change of body roundness index; ΔWHtR, dynamic change of waist-to-height ratio; ΔTyG, dynamic change of triglyceride glucose index; ΔCI, dynamic change of conicity index; ΔABSI, dynamic change of a body shape index; ΔCVAI, dynamic change of Chinese visceral adiposity index.

a Unadjusted.

b Adjusted for age and gender.

c Adjusted for age, gender, place of residence, marital status, educational level, history of smoking and drinking, presence of hypertension, dyslipidemia, systolic blood pressure, diastolic blood pressure, triglycerides, total cholesterol, low-density lipoprotein-cholesterol, high-density lipoprotein-cholesterol, fasting plasma glucose, and hemoglobin A1c at baseline (for ΔTyG, except triglycerides and fasting plasma glucose; for ΔCVAI, except triglycerides and high-density lipoprotein-cholesterol).

The sensitivity analyses suggested that TyG reduction was associated with prediabetes regression to normoglycemia (Supplementary Tables S3, S5). However, the reduction of BRI, WHtR, and CI also showed a significant association with prediabetes regression to normoglycemia. In the subgroup analyses, TyG reduction was associated with prediabetes regression to normoglycemia in all the subgroups while the reduction of other indices was not, which was consistent with the primary analyses (Supplementary Figures S2, S4, S6, S8).

### Attributable fractions of obesity-related indices

The AFs of obesity-related indices and their dynamic changes are shown in Table 4. We obtained the cutoff values by ROC curves, and they were 4.374 for BRI, 0.568 for WHtR, 8.621 for TyG, 1.320 for CI, 0.083 for ABSI, and 106.152 for CVAI, respectively (Supplementary Table S6, Supplementary Figure S9). Low initial BRI (BRI < 4.374), WHtR (WHtR < 0.568), and CVAI (CVAI < 107.794), as well as TyG reduction (ΔTyG < 0) had significant effects on prediabetes regression (BRI: AF 21.10%, 95%CIs: 10.14–32.07%; WHtR: AF 20.85%, 95%CIs: 9.91–31.80%; CVAI, AF 17.48%, 95%CIs: 7.60–27.36%; ΔTyG: AF 17.55%, 95%CIs: 10.25–24.85%), while low initial TyG and the reduction of BRI, WHtR, CI, ABSI, and CVAI showed no significant association with prediabetes regression.

**Table 4:** Attributable fractions of obesity-related indices.

**Variables**	**Model 1** ^**a**^		**Model 2** ^**b**^		**Model 3** ^**c**^	
	**AF% (95%CIs)**	***P*** **-value**	**AF% (95%CIs)**	***P*** **-value**	**AF% (95%CIs)**	***P*** **-value**
BRI < 4.734	28.07 (18.03, 38.11)	< 0.001	28.99 (19.40, 38.59)	< 0.001	21.10 (10.14, 32.07)	< 0.001
WHtR < 0.568	27.75 (17.72, 37.78)	< 0.001	28.70 (19.12, 38.28)	< 0.001	20.85 (9.91, 31.80)	< 0.001
TyG < 8.621	7.55 (0.53, 14.58)	0.035	8.89 (2.21, 15.56)	0.009	6.26 (−2.15, 14.68)	0.144
CI < 1.320	23.37 (13.97, 32.77)	< 0.001	18.11 (7.77, 28.47)	< 0.001	9.57 (−1.61, 20.74)	0.093
ABSI < 0.083	13.57 (6.04, 21.10)	< 0.001	9.26 (1.17, 17.36)	0.025	5.99 (−2.13, 14.10)	0.148
CVAI < 106.152	23.52 (15.05, 31.99)	< 0.001	21.14 (12.41, 29.88)	< 0.001	13.53 (3.43, 23.63)	0.008
ΔBRI < 0	3.28 (−3.03, 9.59)	0.308	3.05 (−3.26, 9.37)	0.343	5.62 (−0.41, 11.65)	0.068
ΔWHtR < 0	3.28 (−3.03, 9.59)	0.308	3.05 (−3.26, 9.37)	0.343	5.62 (−0.41, 11.65)	0.068
ΔTyG < 0	18.32 (11.63, 26.19)	< 0.001	18.61 (11.32, 25.90)	< 0.001	17.55 (10.25, 24.85)	< 0.001
ΔCI < 0	−0.07 (−6.63, 6.49)	0.894	1.04 (−5.47, 7.54)	0.755	2.88 (−3.42, 9.18)	0.37
ΔABSI < 0	−2.99 (−9.75, 3.76)	0.385	−2.07 (−8.76, 4.63)	0.545	−0.23 (−6.73, 6.27)	0.945
ΔCVAI < 0	0.09 (−5.41, 5.50)	0.974	0.36 (−5.15, 5.87)	0.898	2.21 (−3.07, 7.48)	0.413

AF, attributable fraction.

Abbreviations are the same as presented in Tables 2, 3.

a Unadjusted.

b Adjusted for age and gender.

c Adjusted for age, gender, place of residence, marital status, educational level, history of smoking and drinking, presence of hypertension, dyslipidemia, systolic blood pressure, diastolic blood pressure, total cholesterol, low-density lipoprotein-cholesterol, high-density lipoprotein-cholesterol, and hemoglobin A1c at baseline (for TyG and ΔTyG, except triglycerides and fasting plasma glucose; for CVAI and ΔCVAI, except triglycerides and high-density lipoprotein-cholesterol).

**Figure 2:**
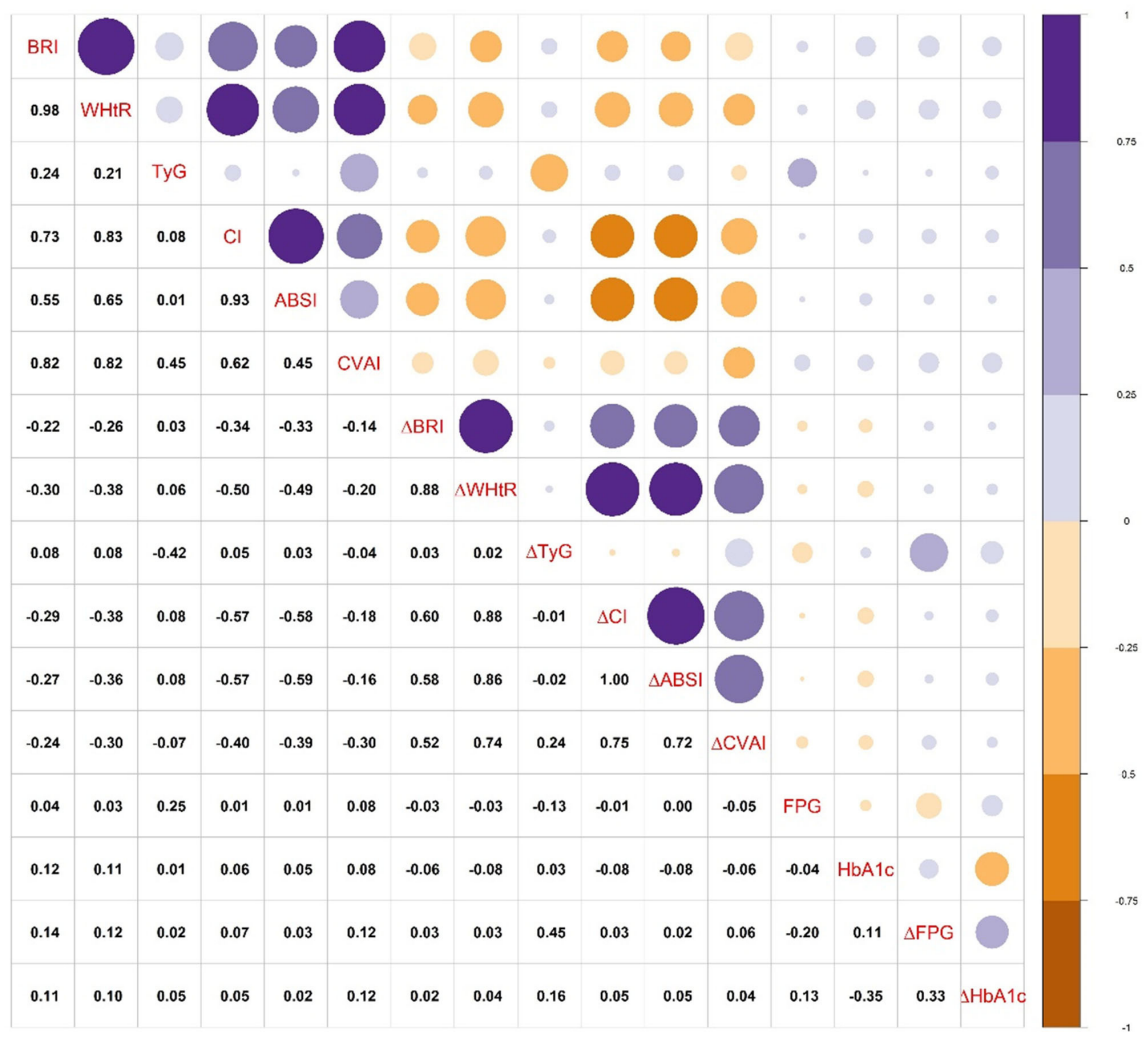
The correlations between obesity-related indices, fasting glucose, and HbA1c. Abbreviations are the same as presented in Tables 2, 3. ΔFPG, dynamic change of FPG; ΔHbA1c, dynamic change of HbA1c.

The correlations between obesity-related indices, fasting glucose, and HbA1c are shown in Figure 2. The initial BRI, WHtR, and CVAI had a stronger correlation with the dynamic changes in FPG (ΔFPG) and HbA1c (ΔHbA1c) than other initial indices. ΔTyG had a stronger correlation with the dynamic changes in ΔFPG and ΔHbA1c than other indices. In addition, BRI, WHtR, CI, and CVAI showed a strong correlation with each other. ΔBRI, ΔWHtR, ΔCI, ΔABSI, and ΔCVAI showed a strong correlation with each other but a weak correlation with ΔTyG.

## Discussion

In the present study, we investigated the association of six obesity-related indices and their dynamic changes with prediabetes regression to normoglycemia among Chinese adults aged ≥45 years during a 4-year follow-up. Our results suggested that low initial BRI, WHtR, and CVAI, as well as TyG reduction were positively associated with the prediabetes regression. Furthermore, the AFs of low initial BRI, WHtR, and CVAI, as well as TyG reduction were all around 20%.

In the present study, 562 (21.61%) participants with prediabetes regressed to normoglycemia. Similarly, the KORA S4 study found that 27.3% of participants reversed from HbA1c-defined prediabetes to normal HbA1c levels, while only 9.3% of participants regressed from glucose-defined prediabetes to NGT based on ADA criteria (15). Moreover, a 10-year follow-up study in Japan reported that 17.1% of participants with prediabetes regressed to normal glucose tolerance (12). The different rates of prediabetes regression may be due to the different definitions of prediabetes, study population, and follow-up years. In the present study, prediabetes was defined using HbA1c and FPG according to the ADA criteria, while in the KORA F4 cohort study (15), prediabetes and normoglycemia were defined only using HbA1c or blood glucose. Furthermore, various characteristics of participants may also explain the different results. Participants aged 45 years and above were included in our study and most of them came from Chinese rural areas, which suggested that the lifestyle of our participants may be different from other study populations.

Abdominal obesity was suggested as a risk of diabetes and may prevent prediabetes regression to normoglycemia (31). BRI, WHtR, and CVAI were recognized as good markers of abdominal fat (18). Although several studies focused on the association of several obesity-related indices with diabetes (32–34), studies on their relationship with prediabetes are lacking. Liu et al. (20) reported that people with higher BRI and WHtR showed an increased risk of developing diabetes. Cai et al. (33) reported that increases in BRI and WHtR were related to an elevated risk of diabetes among Chinese older adults. CVAI, an obesity-related index based on the Chinese population, showed a positive association with diabetes and metabolic abnormality (19). De et al. (35) reported that people who regressed from prediabetes to normoglycemia had lower WHtR than people who did not regress. The present study demonstrated that low initial levels of BRI, WHtR and CVAI were positively associated with prediabetes regression, which indicated that less abdominal fat may contribute to prediabetes regression to normoglycemia. The abdominal fat, a marker of excess ectopic fat, has more metabolically activity than subcutaneous fat. It can secrete a variety of lipoxins associated with metabolic abnormality, hyperinsulinemia, and impaired insulin secretion, damage pancreatic β-cells, increase insulin resistance, and enhance inflammatory responses, thus increasing the risk of diabetes (36).

Notably, the initial CI and ABSI, as well as the reduction of BRI, WHtR, CI, and CVAI also showed significant association with prediabetes regression in the sensitivity analyses based on WHO criteria and the sensitivity analyses based on multinomial logistic regression, which were inconsistent with the results based on ADA criteria. On the one hand, it may be due to the WHO criteria having higher FPG levels to meet prediabetes and do not offer any recommendation on HbA1c criteria for prediabetes. Therefore, fewer people were diagnosed with prediabetes, and a high rate of prediabetes regression to normoglycemia was presented. On the other hand, we made the progression to the diabetes group as the reference in multinomial logistic regression, and the results showed that prediabetes participants with lower initial obesity-related indices and/or the reduction of obesity-related indices contribute to regress to normoglycemia. These sensitivity analysis results suggested lower initial obesity-related indices and/or the reduction of obesity-related indices contribute to a better health outcome among people with prediabetes. However, a longer follow-up period is needed to further identify whether BRI and WHtR reduction contribute to prediabetes regression. For the CI and ABSI showed no significant association with prediabetes in the primary analysis, there may be other explanations besides the diagnostic criteria and analytical methods above. Previous studies reported that CI and ABSI had a low ability to identify prediabetes status (25). In addition, ABSI, which had little correlation with height, weight, or BMI, was originally established to predict mortality in a follow-up cohort, and it may be not a good index to evaluate obesity status (37). CI was highly related to ABSI, and our study also suggested that they had very weak correlations with FPG, HbA1c, and their dynamic changes. Therefore, the two indices may not be good indices to evaluate prediabetes regression.

In the present study, we demonstrated that TyG reduction contributed to prediabetes regression. TyG, calculated by FPG and TG and developed by Simental–Mendia et al. (38), is recognized as a reliable marker for IR (21). Some evidence indicated that its discriminatory ability for IR was better than that of the HOMA-IR and TyG, specifically reflected muscle-related IR (22, 23, 39). Zhang et al. found that increasing TyG elevated the cumulative increased risk of incident diabetes after 6 years of follow-up among 5,706 Chinese rural participants (40). Previous studies reported that TG increment accelerated the progression of prediabetes to diabetes, while TG reduction helped patients with prediabetes regress to normoglycemia (35, 41). In addition, high TG level in the blood has been proven to inhibit insulin activity in muscle and interfere with glucose uptake, while TG overload in islets would impair the function of β cells (42). When both fatty acids and glucose are elevated, the accumulation of metabolites derived from fatty-acid esterification impaired the function of pancreatic β cells (21, 42). Previous research suggested that the capacity for insulin secretion and IR was closely affected by TG and FPG levels (43). Improved pancreatic β-cell function and insulin sensitivity have been shown to increase the odds of reaching normal glucose regulation from prediabetes (31). Our study demonstrated that TyG reduction promoted the progress of prediabetes regression to normoglycemia.

In addition, we further investigated the AFs of obesity-related indices for prediabetes regression. Previous research reported PAFs for diabetes attributable to being overweight, which suggests increases in BMI were responsible for 58% of type 2 diabetes globally. Although several studies have explored the influencing factors of regression from prediabetes to normoglycemia (5, 13, 15), the AFs of the predictors of regression from prediabetes to normoglycemia are lacking. In the present study, the AFs of obesity-related indices were around 20%, which suggested that around 20% of cases regressed to normoglycemia would be attributable to low initial BRI, WHtR, CVAI, or TyG reduction. As a large number of middle-aged and older adults suffered from prediabetes in China (6), the present study suggested that many people with prediabetes would benefit from the prevention of abdominal obesity or the reduction of IR.

The strengths of this study include a prospective cohort study design with a large sample of Chinese middle-aged and older adults. Furthermore, six obesity-related indices have been adopted to access their association with prediabetes regression. In addition, we calculated the AFs of the obesity-related indices and their dynamic changes to make their contributions to prediabetes regression clear.

There are some limitations in this study that should be acknowledged. First, the CHARLS included adults aged 45 years and older from 28 provinces, and the generalization of our findings may not be applicable to all Chinese adults. Second, although our study has controlled for several confounders, some unmeasured variables, such as dietary habits, cardiorespiratory fitness, and metformin use were not included. Third, despite FPG and HbA1c having been used to define prediabetes, the lack of 2 h of post-prandial glucose data from the oral glucose tolerance test may bias the results reported. However, the same criteria have been used in the previous study (7), and the sensitivity analyses also showed consistent results.

In conclusion, our study suggested that low initial BRI, WHtR, and CVAI, as well as TyG reduction, were associated with increased odds of regression to normoglycemia among Chinese middle-aged and older adults with prediabetes. Furthermore, the TyG index can be used as a long-term observation index since its dynamic reduction is beneficial to prediabetes regression. In addition, this study provides more evidence showing that initial obesity-related indices, especially their cutoff values, could be used to identify individuals with prediabetes who are likely to return to normoglycemia. Given that it is difficult for those with prediabetes and a high obesity-related index to return to normoglycemia, more effective lifestyle interventions are warranted to control the conditions in this population.

## Data availability statement

The original contributions presented in the study are included in the article/Supplementary material, further inquiries can be directed to the corresponding authors.

## Author contributions

HY, MinjZ, and JN contributed to the data acquisition, analysis, and results explanation. MinzZ and GL contributed to the results explanation. HY, QH, and RC drafted the manuscript. HY and QH revised the manuscript. All authors have read and agreed to the published version of the manuscript.
