# Supplementary material for: Associations of obesity-related indices with prediabetes regression to normoglycemia among Chinese middle-aged and older adults: a prospective study
Source: Front Nutr. 2023 May 19;10:1075225. doi: 10.3389/fnut.2023.1075225 (PMC10235473; doi:10.3389/fnut.2023.1075225)
Supplement: Supplementary file 1 [file Data_Sheet_1.docx]

# Supplementary Materials

Supplementary Materials Table S1 Association of BMI, WC and their dynamic changes with prediabetes regression

| Variables | Model 1^a^ | | Model 2^b^ | | Model 3^c^ | |
| --- | --- | --- | --- | --- | --- | --- |
|  | OR (95% CIs) | P value | OR (95% CIs) | P value | OR (95% CIs) | P value |
| BMI (per 1) | 0.961(0.936,0.985) | 0.002 | 0.955(0.929,0.980) | 0.001 | 0.978(0.945,1.009) | 0.166 |
| ∆BMI (per 1) | 0.997(0.981,1.003) | 0.501 | 0.997(0.982,1.004) | 0.569 | 0.998(0.981,1.005) | 0.686 |
| WC (per 1) | 0.984(0.977,0.991) | <0.001 | 0.984(0.977,0.290) | <0.001 | 0.989(0.981,0.998) | 0.011 |
| ∆WC (per 1) | 0.992(0.985,0.998) | 0.031 | 0.990(0.986,0.998) | 0.042 | 0.996(0.989,1.000) | 0.181 |

BMI, body mass index; ∆BMI, dynamic change of body mass index; WC, waist circumference; ∆WC, dynamic change of waist circumference.

a Unadjusted.

b Adjusted for age and gender.

c Adjusted for age, gender, place of residence, marital status, educational levels, history of smoking and drinking, presence of hypertension, dyslipidemia, systolic blood pressure, diastolic blood pressure, triglycerides, total cholesterol, low-density lipoprotein-cholesterol, high-density lipoprotein-cholesterol, fasting plasma glucose and hemoglobin A1c at baseline

Supplementary Materials Table S2 Sensitivity upon WHO criteria of initial obesity-related indices and prediabetes regression

|  | No. of cases/total | Model 1^a^ | Model 2^b^ | Model 3^c^ |  |
| --- | --- | --- | --- | --- | --- |
| Variables |  | OR (95% CIs) | OR (95% CIs) | OR (95% CIs) |  |
| BRI |  |  |  |  |  |
| Tertile 1(<3.670) | 254/325 | 2.63(1.87,3.71) | 2.89(2.00,4.21) | 2.35(1.54,3.61) |  |
| Tertile 2(3.670~4.970) | 217/327 | 1.45(1.05,1.99) | 1.52(1.09,2.12) | 1.31(0.91,1.88) |  |
| Tertile 3(>4.970) | 188/326 | 1(Ref.) | 1(Ref.) | 1(Ref.) |  |
| P for trend |  | <0.001 | <0.001 | <0.001 |  |
| WHtR |  |  |  |  |  |
| Tertile 1(<0.516) | 255/326 | 2.62(1.87,3.71) | 2.89(2.00,4.20) | 2.34(1.53,3.59) |  |
| Tertile 2(0.516~0.579) | 215/325 | 1.43(1.04,1.96) | 1.49(1.07,2.09) | 1.29(0.90,1.85) |  |
| Tertile 3(>0.579) | 189/327 | 1(Ref.) | 1(Ref.) | 1(Ref.) |  |
| P for trend |  | <0.001 | <0.001 | <0.001 |  |
| TyG |  |  |  |  |  |
| Tertile 1（<8.579） | 232/326 | 1.33(0.95,1.85) | 1.35(0.97,1.89) | 1.32(0.79,2.21) |  |
| Tertile 2(8.579~9.114) | 215/326 | 1.04(0.75,1.44) | 1.08(0.78,1.50) | 1.21(0.78,1.87) |  |
| Tertile 3(>9.114) | 212/326 | 1(Ref.) | 1(Ref.) | 1(Ref.) |  |
| P for trend |  | 0.095 | 0.077 | 0.301 |  |
| CI |  |  |  |  | |
| Tertile 1(<1.260) | 251/325 | 2.34(1.67,3.29) | 2.29(1.62,3.28) | 1.92(1.32,2.82) | |
| Tertile 2(1.260~1.327) | 212/322 | 1.33(0.97,1.83) | 1.31(0.94,1.82) | 1.10(0.78,1.56) | |
| Tertile 3(>1.327) | 196/331 | 1(Ref.) | 1(Ref.) | 1(Ref.) | |
| P for trend | <0.001 | <0.001 | <0.001 | <0.001 | |
| ABSI |  |  |  |  | |
| Tertile 1(<0.081) | 242/326 | 1.83(1.31,2.56) | 1.75(1.24,2.51) | 1.63(1.12,2.37) | |
| Tertile 2(0.081~0.085) | 217/325 | 1.28(0.93,1.76) | 1.24(0.89,1.73) | 1.13(0.80,1.61) | |
| Tertile 3(>0.085) | 200/327 | 1(Ref.) | 1(Ref.) | 1(Ref.) | |
| P for trend |  | <0.001 | 0.002 | 0.009 | |
| CVAI |  |  |  |  | |
| Tertile 1(<84.823) | 245/326 | 2.34(1.68,3.28) | 2.29(1.64,3.22) | 2.02(1.34,3.05) | |
| Tertile 2(84.823~120.946) | 223/325 | 1.89(1.37,2.62) | 1.85(1.34,2.58) | 1.70(1.18,2.44) | |
| Tertile 3(>120.946) | 191/327 | 1(Ref.) | 1(Ref.) | 1(Ref.) | |
| P for trend |  | <0.001 | <0.001 | <0.001 | |

Abbreviations are the same as in Table 2

a Unadjusted.

b Adjusted for age and gender.

c Adjusted for age, gender, place of residence, marital status, educational levels, history of smoking and drinking, presence of hypertension, dyslipidemia, systolic blood pressure, diastolic blood pressure, triglycerides, total cholesterol, low-density lipoprotein-cholesterol, high-density lipoprotein-cholesterol, fasting plasma glucose and hemoglobin A1c at baseline (for TyG, except triglycerides and fasting plasma glucose; for CVAI, except triglycerides and high-density lipoprotein-cholesterol).

| Variables | No. of cases/total | Model 1^a^ | Model 2^b^ | Model 3^c^ |
| --- | --- | --- | --- | --- |
|  |  | OR (95% CIs) | OR (95% CIs) | OR (95% CIs) |
| ∆BRI |  |  |  |  |
| Decreased (<-0.240) | 231/326 | 1.78(1.26,2.53) | 1.60(1.15,2.23) | 1.78(1.26,2.53) |
| Stable (-0.240~0.434) | 229/326 | 1.63(1.15,2.30) | 1.52(1.10,2.11) | 1.63(1.15,2.30) |
| Increased (>0.434) | 199/326 | 1(Ref.) | 1(Ref.) | 1(Ref.) |
| P for trend |  | 0.008 | 0.005 | 0.001 |
| ∆WHtR |  |  |  |  |
| Decreased (<-0.012) | 231/326 | 1.45(1.05,2.02) | 1.50(1.08,2.09) | 1.70(1.20,2.42) |
| Stable (-0.012~0.020) | 224/326 | 1.31(0.95,1.82) | 1.34(0.96,1.85) | 1.46(1.04,2.07) |
| Increased (>0.020) | 204/326 | 1(Ref.) | 1(Ref.) | 1(Ref.) |
| P for trend |  | 0.024 | 0.015 | 0.003 |
| ∆TyG |  |  |  |  |
| Decreased(<-0.273） | 275/325 | 5.43(3.77,7.94) | 5.54(3.83,8.11) | 7.34(4.85,11.32) |
| Stable (-0.273~0.207) | 220/327 | 2.03(1.48,2.79) | 2.07(1.51,2.85) | 2.33(1.66,3.28) |
| Increased (>0.207) | 164/326 | 1(Ref.) | 1(Ref.) | 1(Ref.) |
| P for trend |  | <0.001 | <0.001 | <0.001 |
| ∆CI |  |  |  |  |
| Decreased(T1, <-0.026） | 231/326 | 1.36(0.98,1.89) | 1.39(1.01,1.94) | 1.52(1.07,2.15) |
| Stable (-0.026~0.037) | 219/326 | 1.15(0.83,1.58) | 1.15(0.83,1.93) | 1.18(0.82,1.62) |
| Increased (>0.037) | 209/326 | 1(Ref.) | 1(Ref.) | 1(Ref.) |
| P for trend |  | 0.066 | 0.049 | 0.020 |
| ∆ABSI |  |  |  |  |
| Decreased(<-0.002） | 233/325 | 1.49(1.08,2.08) | 1.52(1.09,2.12) | 1.64(1.16,2.33) |
| Stable (-0.002~0.002) | 221/327 | 1.23(0.89,1.70) | 1.22(0.88,1.69) | 1.23(0.87,1.73) |
| Increased (>0.002) | 205/326 | 1(Ref.) | 1(Ref.) | 1(Ref.) |
| P for trend |  | 0.017 | 0.013 | 0.006 |
| ∆CVAI |  |  |  |  |
| Decreased(<-3.958） | 229/325 | 1.53(1.10,2.13) | 1.53(1.14,2.21) | 1.75(1.23,2.50) |
| Stable (-3.958~14.268) | 224/327 | 1.35(0.98,1.87) | 1.42(1.03,1.98) | 1.48(1.05,2.10) |
| Increased (>14.268) | 206/326 | 1(Ref.) | 1(Ref.) | 1(Ref.) |
| P for trend |  | 0.011 | 0.006 | 0.002 |

Supplementary Materials Table S3 Sensitivity upon WHO criteria of dynamic changes of obesity-related indices and prediabetes regression

Abbreviations are the same as in Table 3

a Unadjusted.

b Adjusted for age and gender.

c Adjusted for age, gender, place of residence, marital status, educational levels, history of smoking and drinking, presence of hypertension, dyslipidemia, systolic blood pressure, diastolic blood pressure, triglycerides, total cholesterol, low-density lipoprotein-cholesterol, high-density lipoprotein-cholesterol, fasting plasma glucose and hemoglobin A1c at baseline (for ∆TyG, except triglycerides and fasting plasma glucose; for ∆CVAI, except triglycerides and high-density lipoprotein-cholesterol).

Supplementary Materials Table S4 Sensitivity upon multinomial logistic regression of dinitial obesity-related indices and prediabetes regression ^a^

| Variables | remain as prediabetes |  | regression to normoglycemia |
| --- | --- | --- | --- |
|  | OR (95% CIs) |  | OR (95% CIs) |
| BRI |  |  |  |
| Tertile 1(<3.582) | 2.00(1.44,2.77) |  | 2.58(1.74,3.82) |
| Tertile 2(3.582~4.818) | 1.39(1.05,1.83) |  | 1.79(1.27,2.54) |
| Tertile 3(>4.818) | 1(Ref.) |  | 1(Ref.) |
| P for trend | <0.001 |  | <0.001 |
| WHtR |  |  |  |
| Tertile 1(<0.512) | 2.00(1.44,2.77) |  | 2.60(1.75,3.85) |
| Tertile 2(0.512~0.572) | 1.39(1.05,1.83) |  | 1.82(1.75,2.57) |
| Tertile 3(>0.572) | 1(Ref.) |  | 1(Ref.) |
| P for trend | <0.001 |  | <0.001 |
| TyG |  |  |  |
| Tertile 1（<8.463） | 1.28(0.82,1.98) |  | 1.40(0.82,2.38) |
| Tertile 2(8.463~8.934) | 0.99(0.70,1.41) |  | 1.10(0.72,0.69) |
| Tertile 3(>8.934) | 1(Ref.) |  | 1(Ref.) |
| P for trend | 0.233 |  | 0.209 |
| CI |  |  |  |
| Tertile 1（< 1.254） | 1.42(1.05,1.92) |  | 1.60(1.12,2.30) |
| Tertile 2(1.254~1.324) | 1.16(0.88,1.52) |  | 1.35(0.96,1.89) |
| Tertile 3(>1.324) | 1(Ref.) |  | 1(Ref.) |
| P for trend | 0.022 |  | 0.013 |
| ABSI |  |  |  |
| Tertile 1（<0.081） | 1.27(0.95,1.70) |  | 1.17(0.82,1.67) |
| Tertile 2(0.081~0.085) | 1.26(0.96,1.66) |  | 1.23(0.87,1.72) |
| Tertile 3(>0.085) | 1(Ref.) |  | 1(Ref.) |
| P for trend | 0.099 |  | 0.364 |
| CVAI |  |  |  |
| Tertile 1（<81.131） | 1.73(1.22,2.46) |  | 2.07(1.36,3.16) |
| Tertile 2 (81.131~114.538) | 1.36(1.02,1.81) |  | 1.60(1.12,2.28) |
| Tertile 3(>114.538) | 1(Ref.) |  | 1(Ref.) |
| P for trend | 0.001 |  | <0.001 |

Abbreviations are the same as in Table 2

a Adjusted for age, gender, place of residence, marital status, educational levels, history of smoking and drinking, presence of hypertension, dyslipidemia, systolic blood pressure, diastolic blood pressure, triglycerides, total cholesterol, low-density lipoprotein-cholesterol, high-density lipoprotein-cholesterol, fasting plasma glucose and hemoglobin A1c at baseline (for TyG, except triglycerides and fasting plasma glucose; for CVAI, except triglycerides and high-density lipoprotein-cholesterol).

Supplementary Materials Table S5 Sensitivity upon multinomial logistic regression of dynamic changes of obesity-related indices and prediabetes regression ^a^

| Variables | remain as prediabetes |  | regression to normoglycemia |
| --- | --- | --- | --- |
|  | OR (95% CIs) |  | OR (95% CIs) |
| ∆BRI |  |  |  |
| Decreased (<-0.240) | 1.89(1.43,2.49) |  | 1.94(1.39,2.72) |
| Stable (-0.240~0.434) | 1.71(1.30,2.25) |  | 1.64(1.17,2.28) |
| Increased (>0.434) | 1(Ref.) |  | 1(Ref.) |
| P for trend | <0.001 |  | <0.001 |
| ∆WHtR |  |  |  |
| Decreased (<-0.012) | 1.93(1.46,2.55) |  | 1.97(1.40,2.76) |
| Stable (-0.012~0.020) | 1.63(1.24,2.13) |  | 1.52(1.09,2.11) |
| Increased (>0.020) | 1(Ref.) |  | 1(Ref.) |
| P for trend | <0.001 |  | <0.001 |
| ∆TyG |  |  |  |
| Decreased(<-0.273） | 5.55(3.97,7.74) |  | 9.29(6.26,13.78) |
| Stable (-0.273~0.207) | 3.11(2.36,4.11) |  | 3.54(2.50,5.01) |
| Increased (>0.207) | 1(Ref.) |  | 1(Ref.) |
| P for trend | <0.001 |  | <0.001 |
| ∆CI |  |  |  |
| Decreased(T1, <-0.026） | 1.66(1.26,2.19) |  | 1.58(1.12,2.22) |
| Stable (-0.026~0.037) | 1.33(1.01,1.74) |  | 1.45(1.05,2.02) |
| Increased (>0.037) | 1(Ref.) |  | 1(Ref.) |
| P for trend | <0.001 |  | 0.006 |
| ∆ABSI |  |  |  |
| Decreased(<-0.002） | 1.45(1.09,1.91) |  | 1.29(0.92,1.81) |
| Stable (-0.002~0.002) | 1.08(0.82,1.41) |  | 1.09(0.78,1.51) |
| Increased (>0.002) | 1(Ref.) |  | 1(Ref.) |
| P for trend | 0.010 |  | 0.144 |
| ∆CVAI |  |  |  |
| Decreased(<-3.958） | 2.10(1.58,2.79) |  | 2.24(1.59,3.16) |
| Stable (-3.958~14.268) | 1.68(1.28,2.22) |  | 1.59(1.14,2.22) |
| Increased (>14.268) | 1(Ref.) |  | 1(Ref.) |
| P for trend | <0.001 |  | <0.001 |

Abbreviations are the same as in Table 3

a Adjusted for age, gender, place of residence, marital status, educational levels, history of smoking and drinking, presence of hypertension, dyslipidemia, systolic blood pressure, diastolic blood pressure, triglycerides, total cholesterol, low-density lipoprotein-cholesterol, high-density lipoprotein-cholesterol, fasting plasma glucose and hemoglobin A1c at baseline (for ∆TyG, except triglycerides and fasting plasma glucose; for ∆CVAI, except triglycerides and high-density lipoprotein-cholesterol).

Supplementary Materials Figure S1 Subgroup analyses stratified by age for the association of initial obesity-related indices with prediabetes regression.


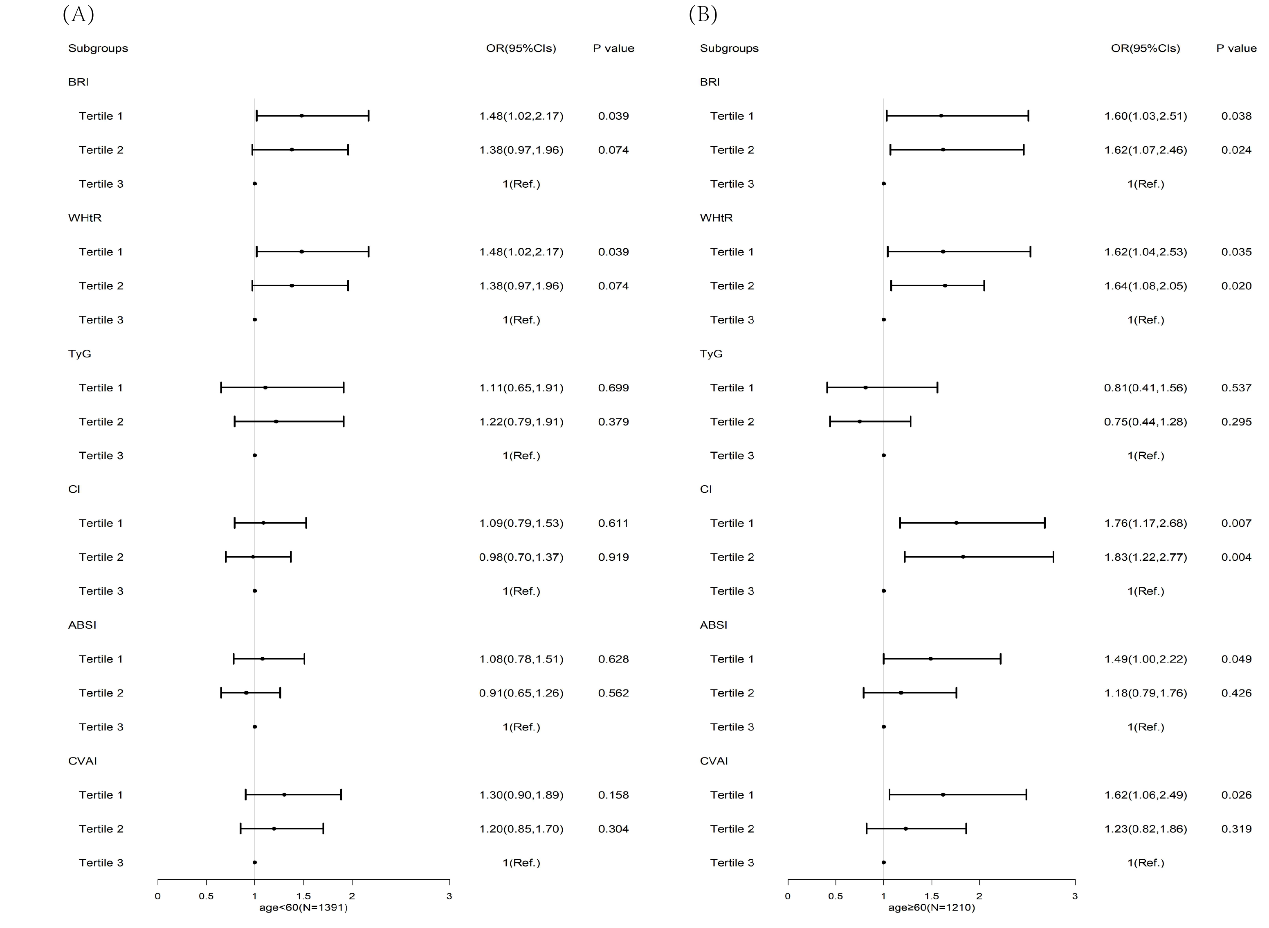


(A) The association of initial obesity-related indices with prediabetes regression among participants < 60 years. (B) The association of initial obesity-related indices with prediabetes regression among participants ≥ 60 years.

Multinomial logistic regression analysis was conducted to obtain the ORs and 95% confidence intervals for the association of initial obesity-related indices in tertiles with prediabetes regression to normoglycemia based on the American Diabetes Association criteria. All the analyses were controlled for age, gender, place of residence, marital status, educational levels, history of smoking and drinking, presence of hypertension and dyslipidemia, systolic blood pressure, diastolic blood pressure, triglycerides, total cholesterol, low-density lipoprotein-cholesterol, high-density lipoprotein-cholesterol and hemoglobin A1c at baseline (for TyG, except triglycerides and fasting plasma glucose; for CVAI, except triglycerides and high-density lipoprotein-cholesterol).

Supplementary Materials Figure S2 Subgroup analyses stratified by age for the association of dynamic changes of obesity-related indices with prediabetes regression.


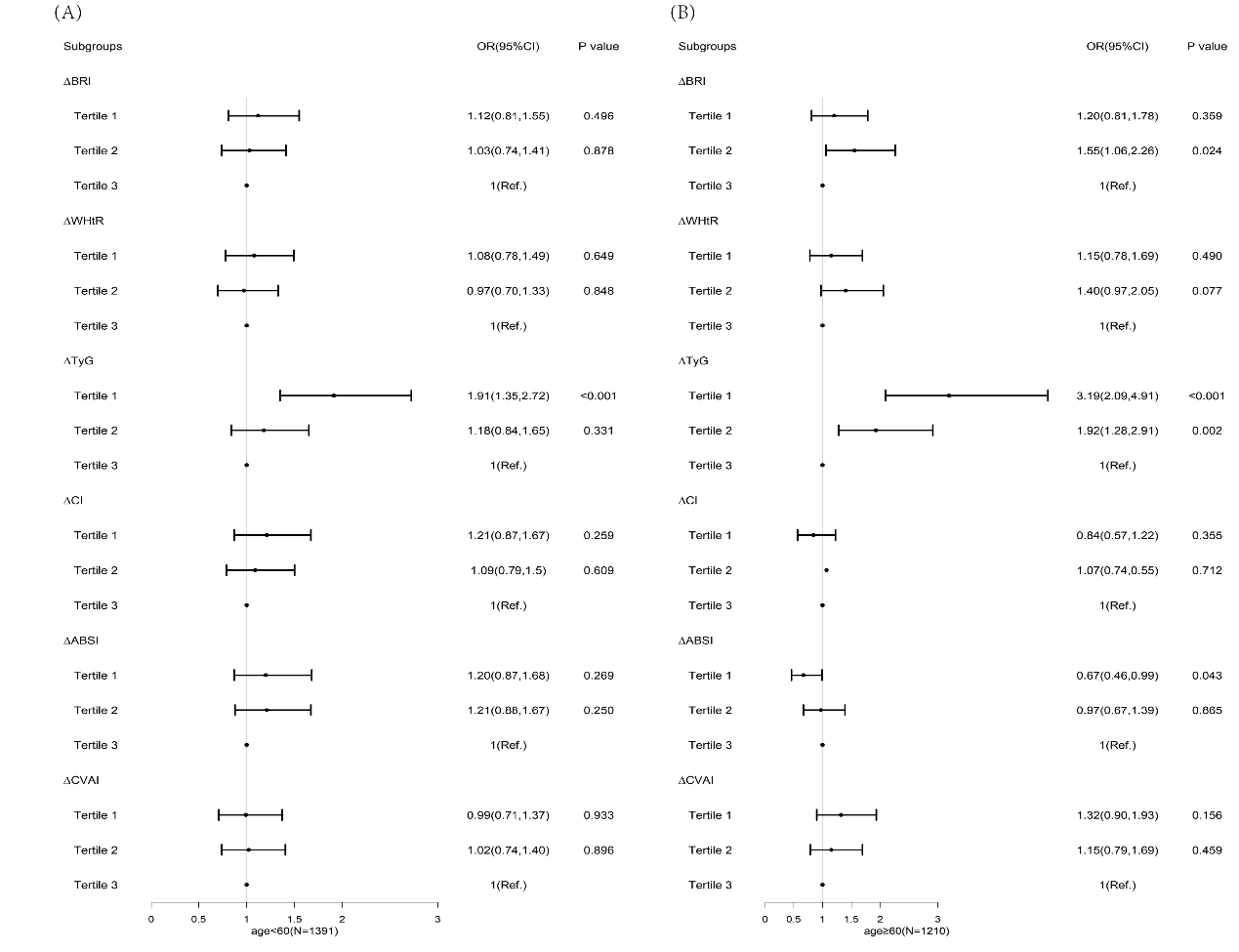


(A) The association of dynamic changes of obesity-related indices with prediabetes regression among participants < 60 years. (B) The association of dynamic changes of obesity-related indices with prediabetes regression among participants ≥ 60 years.

Multinomial logistic regression analysis was conducted to obtain the ORs and 95% confidence intervals for the association of dynamic changes of obesity indices in tertiles with prediabetes regression to normoglycemia based on the American Diabetes Association criteria. All the analyses were controlled for age, gender, place of residence, marital status, educational levels, history of smoking and drinking, presence of hypertension and dyslipidemia, systolic blood pressure, diastolic blood pressure, triglycerides, total cholesterol, low-density lipoprotein-cholesterol, high-density lipoprotein-cholesterol and hemoglobin A1c at baseline (for ∆TyG, except triglycerides and fasting plasma glucose; for ∆CVAI, except triglycerides and high-density lipoprotein-cholesterol).

Supplementary Materials Figure S3 Subgroup analyses stratified by gender for the association of initial obesity-related indices with prediabetes regression.


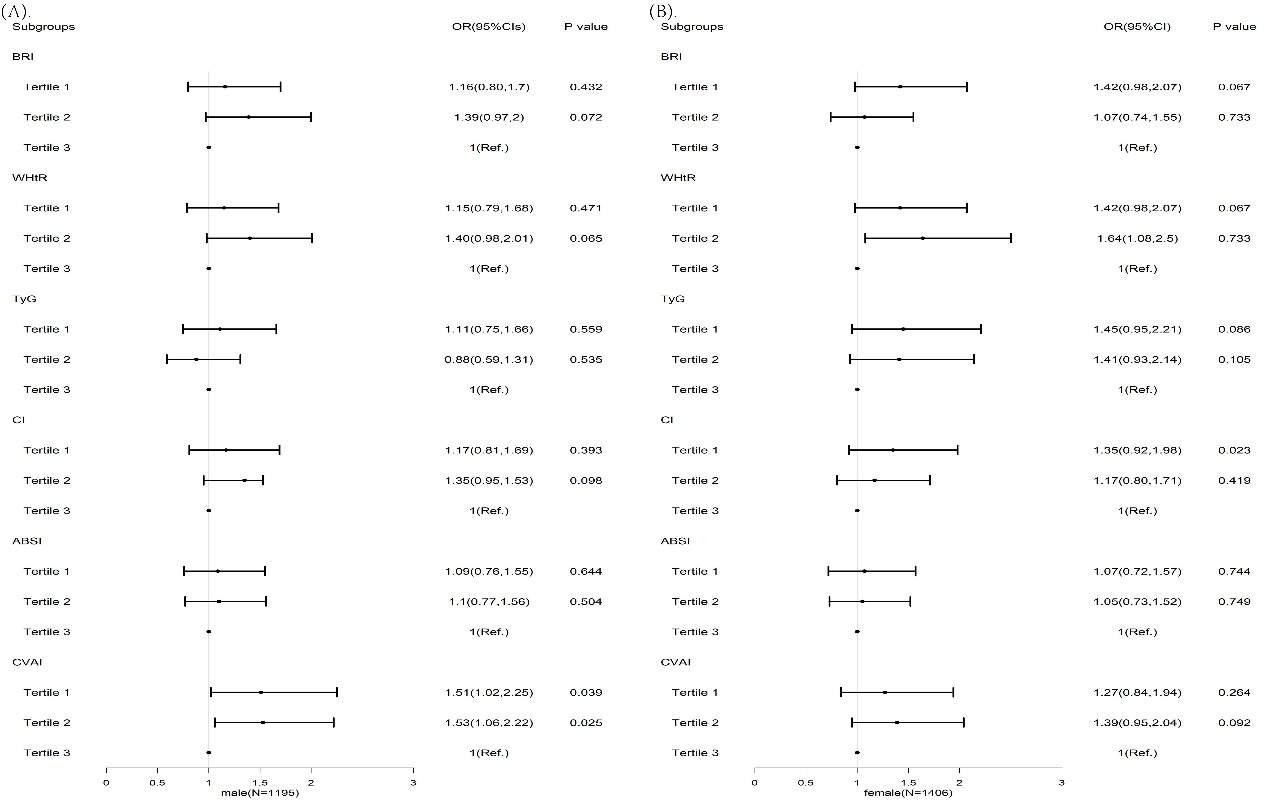


(A) The association of initial obesity-related indices with prediabetes regression among male participants. (B) The association of initial obesity-related indices with prediabetes regression among female participants.

Multinomial logistic regression analysis was conducted to obtain the ORs and 95% confidence intervals for the association of initial obesity indices in tertiles with prediabetes regression to normoglycemia based on the American Diabetes Association criteria. All the analyses were controlled for age, place of residence, marital status, educational levels, history of smoking and drinking, presence of hypertension and dyslipidemia, systolic blood pressure, diastolic blood pressure, triglycerides, total cholesterol, low-density lipoprotein-cholesterol, high-density lipoprotein-cholesterol and hemoglobin A1c at baseline (for ∆TyG, except triglycerides and fasting plasma glucose; for ∆CVAI, except triglycerides and high-density lipoprotein-cholesterol).

Supplementary Materials Figure S4 Subgroup analyses stratified by gender for the association of dynamic changes of obesity-related indices with prediabetes regression.


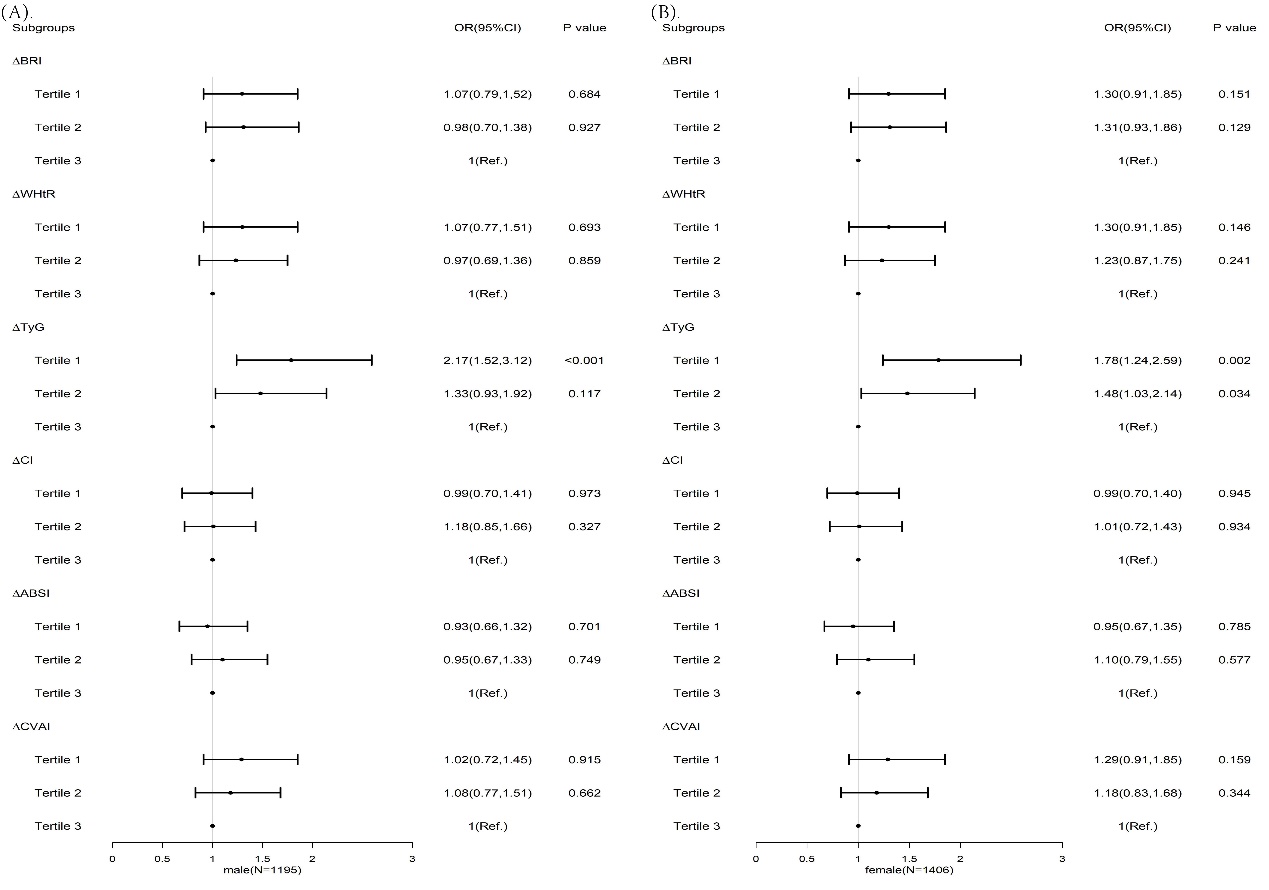


(A) The association of dynamic changes of obesity-related indices with prediabetes regression among male participants. (B) The association of dynamic changes of obesity-related indices with prediabetes regression among female participants.

Multinomial logistic regression analysis was conducted to obtain the ORs and 95% confidence intervals for the association of dynamic changes of obesity indices in tertiles with prediabetes regression to normoglycemia based on the American Diabetes Association criteria. All the analyses were controlled for age, place of residence, marital status, educational levels, history of smoking and drinking, presence of hypertension and dyslipidemia, systolic blood pressure, diastolic blood pressure, triglycerides, total cholesterol, low-density lipoprotein-cholesterol, high-density lipoprotein-cholesterol and hemoglobin A1c at baseline (for ∆TyG, except triglycerides and fasting plasma glucose; for ∆CVAI, except triglycerides and high-density lipoprotein-cholesterol).

Supplementary Materials Figure S5 Subgroup analyses stratified by drinking status for the association of initial obesity-related indices with prediabetes regression.


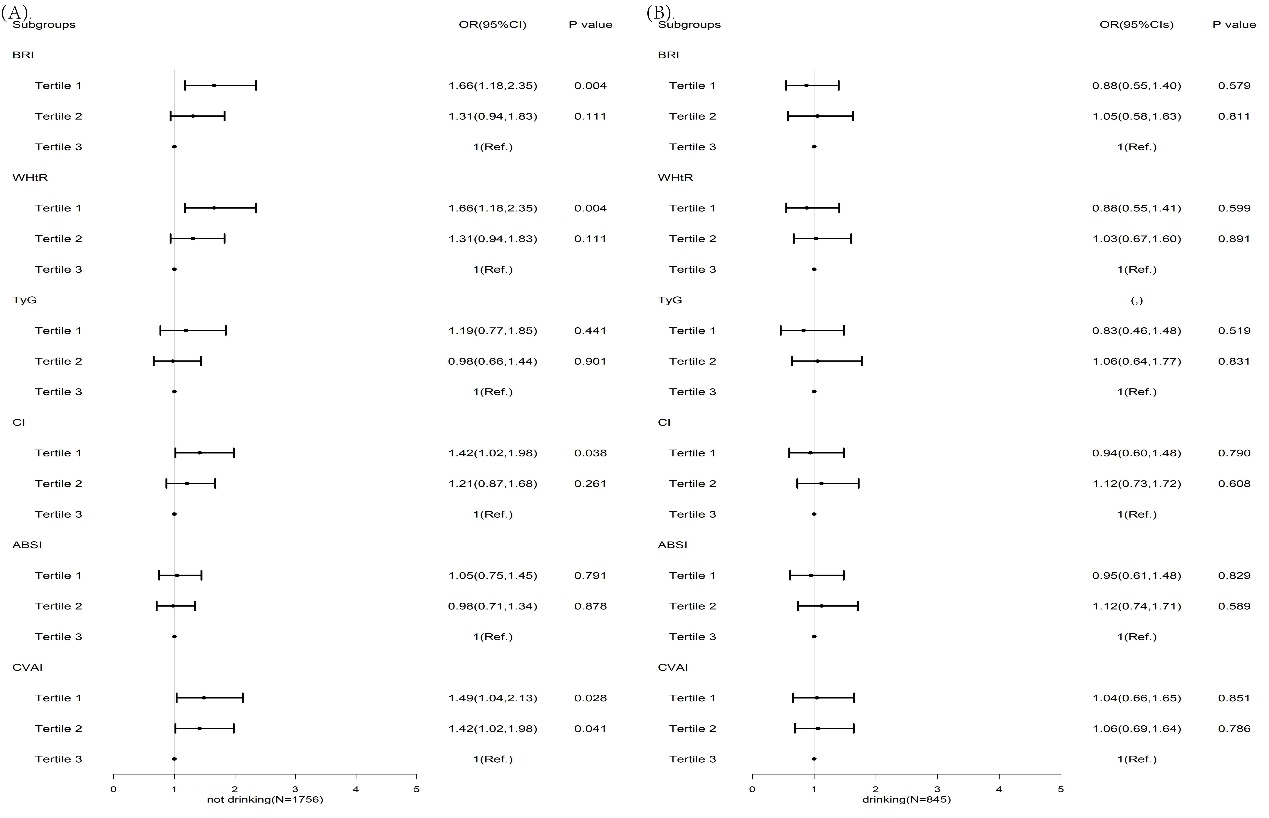


(A) The association of initial obesity-related indices with prediabetes regression among not drinking participants. (B) The association of initial obesity-related indices with prediabetes regression among drinking participants.

Multinomial logistic regression analysis was conducted to obtain the ORs and 95% confidence intervals for the association of initial obesity indices in tertiles with prediabetes regression to normoglycemia based on the American Diabetes Association criteria. All the analyses were controlled for age, gender, place of residence, marital status, educational levels, history of smoking, presence of hypertension and dyslipidemia, systolic blood pressure, diastolic blood pressure, triglycerides, total cholesterol, low-density lipoprotein-cholesterol, high-density lipoprotein-cholesterol and hemoglobin A1c at baseline (for ∆TyG, except triglycerides and fasting plasma glucose; for ∆CVAI, except triglycerides and high-density lipoprotein-cholesterol).

Supplementary Materials Figure S6 Subgroup analyses stratified by drinking status for the association of dynamic changes of obesity-related indices with prediabetes regression.


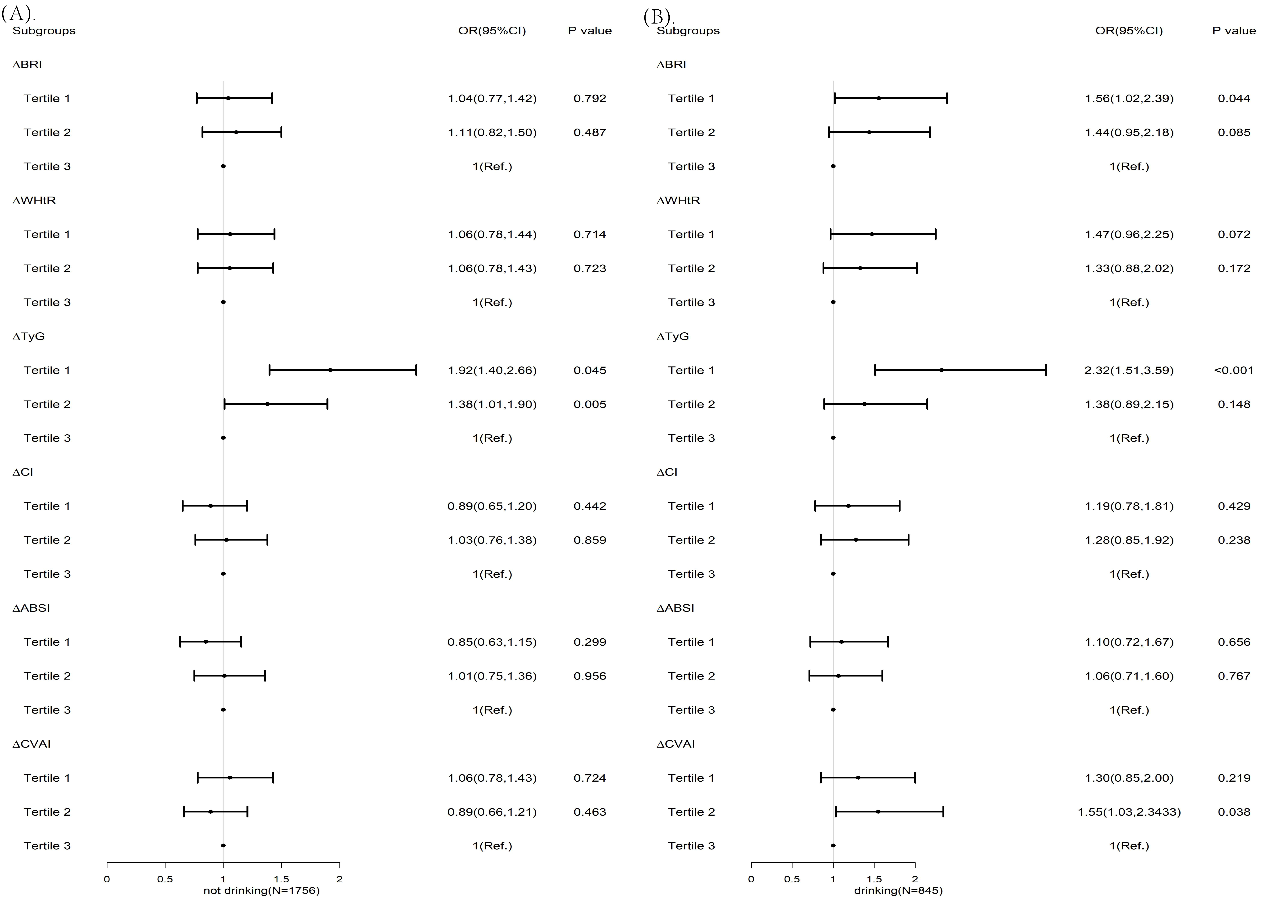


(A) The association of dynamic changes of obesity-related indices with prediabetes regression among not drinking participants. (B) The association of dynamic changes of obesity-related indices with prediabetes regression among drinking participants.

Multinomial logistic regression analysis was conducted to obtain the ORs and 95% confidence intervals for the association of dynamic changes of obesity indices in tertiles with prediabetes regression to normoglycemia based on the American Diabetes Association criteria. All the analyses were controlled for age, gender, place of residence, marital status, educational levels, history of smoking, presence of hypertension and dyslipidemia, systolic blood pressure, diastolic blood pressure, triglycerides, total cholesterol, low-density lipoprotein-cholesterol, high-density lipoprotein-cholesterol and hemoglobin A1c at baseline (for ∆TyG, except triglycerides and fasting plasma glucose; for ∆CVAI, except triglycerides and high-density lipoprotein-cholesterol).

Supplementary Materials Figure S7 Subgroup analyses stratified by initial HbA1c for the association of initial obesity-related indices with prediabetes regression.


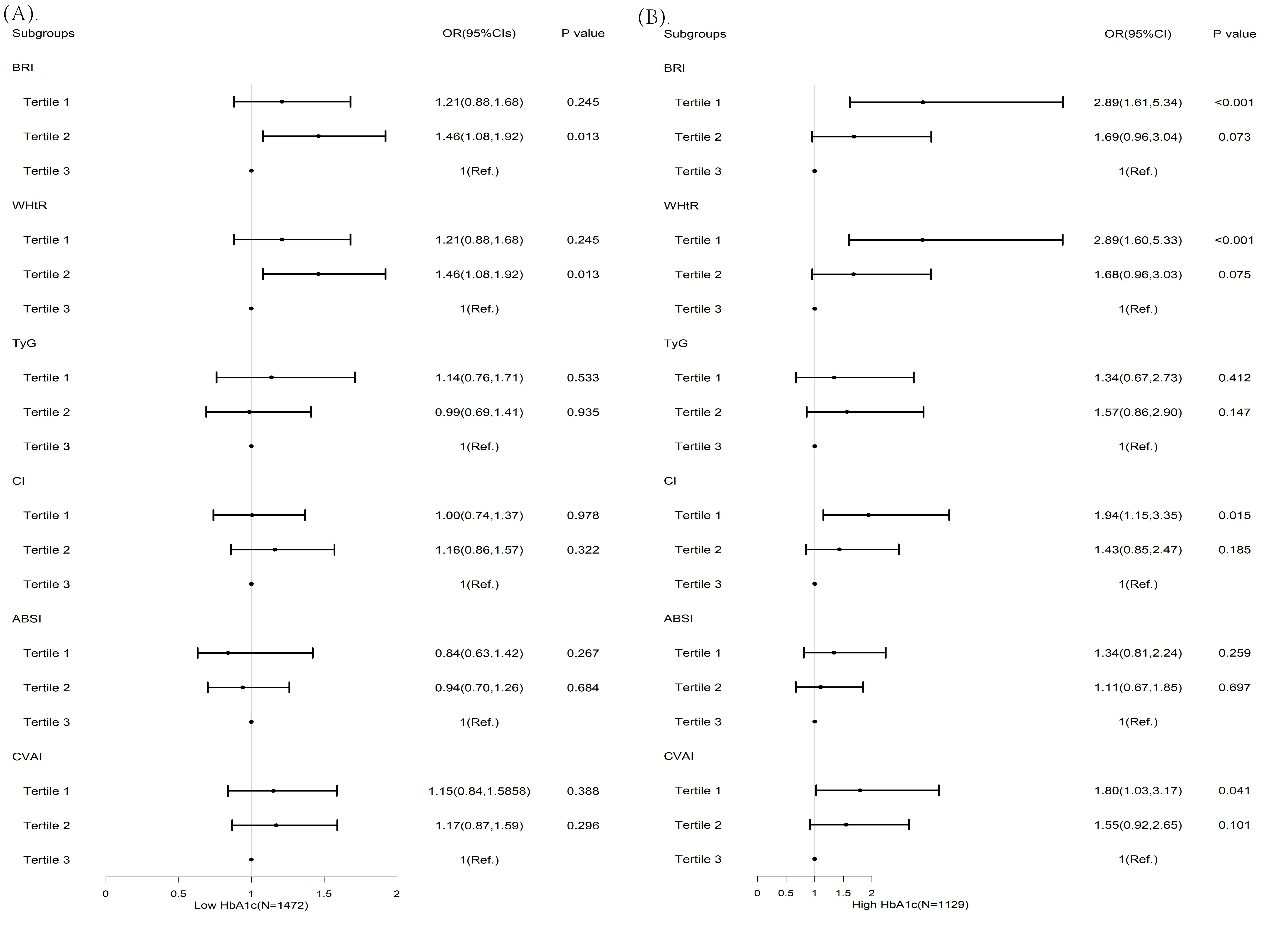


(A) The association of initial obesity-related indices with prediabetes regression among participants with low initial HbA1c level. (B) The association of initial obesity-related indices with prediabetes regression among participants high initial HbA1c level.

Multinomial logistic regression analysis was conducted to obtain the ORs and 95% confidence intervals for the association of initial obesity indices in tertiles with prediabetes regression to normoglycemia based on the American Diabetes Association criteria. All the analyses were controlled for age, gender, place of residence, marital status, educational levels, history of smoking and drinking, presence of hypertension and dyslipidemia, systolic blood pressure, diastolic blood pressure, triglycerides, total cholesterol, low-density lipoprotein-cholesterol, high-density lipoprotein-cholesterol and hemoglobin A1c at baseline (for ∆TyG, except triglycerides and fasting plasma glucose; for ∆CVAI, except triglycerides and high-density lipoprotein-cholesterol).

Supplementary Materials Figure S8 Subgroup analyses stratified by initial HbA1c for the association of dynamic changes of obesity-related indices with prediabetes regression.


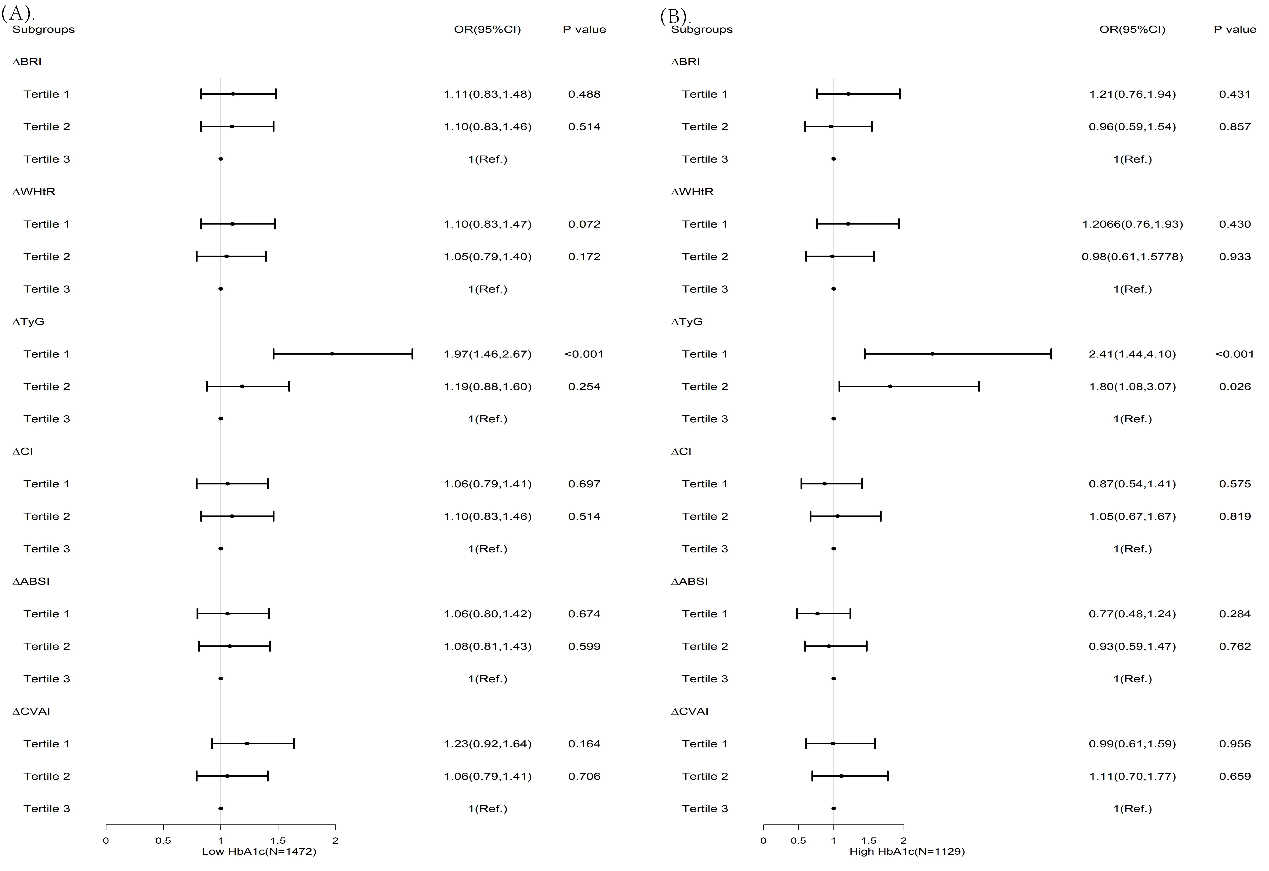


(A) The association of dynamic changes of obesity-related indices with prediabetes regression among participants with low initial HbA1c level. (B) The association of dynamic changes of obesity-related indices with prediabetes regression among participants high initial HbA1c level.

Multinomial logistic regression analysis was conducted to obtain the ORs and 95% confidence intervals for the association of dynamic changes of obesity indices in tertiles with prediabetes regression to normoglycemia based on the American Diabetes Association criteria. All the analyses were controlled for age, gender, place of residence, marital status, educational levels, history of smoking and drinking, presence of hypertension and dyslipidemia, systolic blood pressure, diastolic blood pressure, triglycerides, total cholesterol, low-density lipoprotein-cholesterol, high-density lipoprotein-cholesterol and hemoglobin A1c at baseline (for ∆TyG, except triglycerides and fasting plasma glucose; for ∆CVAI, except triglycerides and high-density lipoprotein-cholesterol).

Table S6. ROC analysis of obesity-related indices and prediabetes regression

|  | AUC (95%CI) | P | Cut-off value | Sensitivity | Specificity | Youden index |
| --- | --- | --- | --- | --- | --- | --- |
| BRI | 0.580(0.555,0.606) | <0.001 | 4.734 | 0.757 | 0.387 | 0.144 |
| WHtR | 0.580(0.555,0.606) | <0.001 | 0.568 | 0.757 | 0.387 | 0.144 |
| TyG | 0.531(0.504,0.559) | 0.023 | 8.621 | 0.562 | 0.502 | 0.064 |
| CI | 0.569(0.543,0.596) | <0.001 | 1.320 | 0.730 | 0.377 | 0.107 |
| ABSI | 0.551(0.525,0.578) | <0.001 | 0.083 | 0.575 | 0.515 | 0.090 |
| CVAI | 0.566(0.540,0.592) | <0.001 | 106.152 | 0.689 | 0.434 | 0.123 |

Abbreviations are the same as in Table S2.

Figure S9. ROC curves of obesity-related indices and prediabetes regression


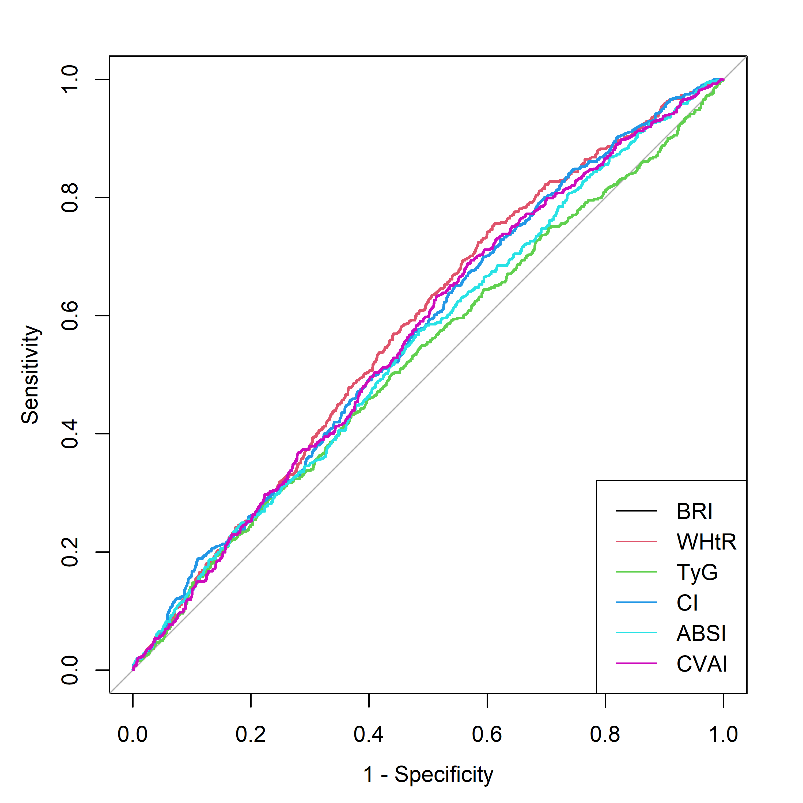


Abbreviations are the same as in Table S2.
